# Morphological and Gamma-Ray Attenuation Properties of High-Density Polyethylene Containing Bismuth Oxide

**DOI:** 10.3390/ma15186410

**Published:** 2022-09-15

**Authors:** Aljawhara H. Almuqrin, Mohamed Elsafi, Sabina Yasmin, M. I. Sayyed

**Affiliations:** 1 Department of Physics, College of Science, Princess Nourah bint Abdulrahman University, P.O. Box 84428, Riyadh 11671, Saudi Arabia; 2 Physics Department, Faculty of Science, Alexandria University, Alexandria 21511, Egypt; 3 Department of Physics, Chittagong University of Engineering and Technology, Chattogram 4349, Bangladesh; 4 Department of Physics, Faculty of Science, Isra University, Amman 11622, Jordan

**Keywords:** HDPE, Bi_2_O_3_, effective atomic number, novel radiation shielding material

## Abstract

For extensive radiation exposure, inventing a novel radiation shielding material is a burning issue at present for the purpose of life saving. Considering this thought, in this study, by adding sundry amounts of Bi_2_O_3_ into pure high-density polyethylene (HDPE), six HDPE systems were prepared to evaluate the radiation shielding efficiency. These HDPE systems were HDPEBi-0 (pure HDPE), HDPEBi-10 (10 wt% Bi_2_O_3_), HDPEBi-20 (20 wt% Bi_2_O_3−_), HDPEBi-30 (30 wt% Bi_2_O_3_), HDPEBi-40 (40 wt% Bi_2_O_3_), and HDPEBi-50 (50 wt% Bi_2_O_3_). The values of the linear attenuation coefficients of the experimental results (calculated in the lab using HPGe) were compared with the theoretical results (obtained using Phy-X software) at 0.060, 0.662, 1.173, and 1.333 MeV energies. To ensure the accurateness of the experimental results, this comparison was made. It was crystal clear that for energy values from 0.06 MeV to 1.333 MeV, all the experimental values were in line with Phy-X software data, which demonstrated the research setup’s reliability. Here, the linear attenuation coefficient (LAC), and mean free path (MFP) shielding parameters were assessed. At the energy of 1.333 MeV, sample HDPEBi-0 showed an HVL value 1.7 times greater than that of HDPEBi-50, yet it was 23 times greater at 0.0595 MeV. That means that for proper radiation protection, very-low-energy HDPE systems containing 10–50% Bi_2_O_3_ could be used; however, the thickness of the HDPE system must be increased according to the energy of incident radiation.

## 1. Introduction

Natural ionizing radiation enduringly eclipses earth [1]. In our modern life, uses of radiation are mandatory in different sectors, such as the use of ionizing radiation in scientific disciplines, X-rays in medical and security checkpoints at airports, and computed tomography scans and radio-therapy in oncology departments [2,3]. To ensure careful control of the radiation received and protect people from unexpected exposure to radiation, shielding is one of the supreme priorities [4,5]. Usually, inorganic glasses [6], metal [7], ceramics [8,9], and organic polymers [10,11] are used for protection against hazardous radiation. Polymers such as polyethylene, polystyrene, polyvinyl chloride, polyacrylates, and polysiloxanes have been taken into account as organic protective materials for the prevention of radiation hazards [12]. The flexibility, durability, and featherweight features of polymer compounds have driven researchers to choose polymers as radiation shielding materials [11]. Bismuth borate glasses have shown healthier radiation protection ability than lead glass and steel–magnetite concrete [13].

The addition of high-density oxides such as PbO, Bi_2_O_3_, and WO_3_ to the matrix material enhances the shielding ability of this material due to the large atomic number of Pb, Bi, and W elements [14]. Although the Bi^+3^ ion has a large density and effective atomic number, it is not yet possible to synthesize glass using individual Bi^+3^ ions. However, glass with added Bi_2_O_3_ is considered one of the most important radiation protective materials [15]. Onuoha et al. researched the mechanical properties of recycled polypropylene composites filled with periwinkle powder. It was found that periwinkle shell powder enhanced the tensile strength, Young’s modulus, and hardness of polypropylene composites [16]. In 2022, Abdolahzadeh et al. investigated the shielding and mechanical properties of HDPE containing nano-tungsten oxide, bismuth oxide, and barium sulfate. The results confirmed that the value of LAC increased with the increase in the amount of filler used [17]. Very recently, in 2020, Lun et al. investigated the tensile properties of polyethylene composites containing geological kaolin as fillers. The obtained results specified that 8% kaolin filler provided the highest tensile properties [18].

Moreover, due to the low rates of crystallization, non-toxicity, high radioactive resistance, large optical basicity, high third-order nonlinear optical susceptibility, high polarizability, long infrared cut-off wavelengths, and moisture resistance, Bi_2_O_3_-containing glass (as a replacement of PbO) are utilized for radiation shielding purposes [19,20,21,22,23]. In fabrics, adding bismuth oxide as the replacement of lead boosts the shielding ability to counter X-rays [3]. That is why the purpose of this research study was to develop the shielding ability of HDPE by accumulating Bi_2_O_3_ into it by taking into account the measuring values of the LAC, HVL, MFP, and Z_eff_ shielding parameters in the energy range from 0.015 MeV to 15 MeV. Additionally, for validating the experimental setup, the values of the linear attenuation coefficients of the prepared high-density polyethylene measured using an HPGe detector were coordinated with the theoretical results obtained using Phy-X software. To the best of the authors’ knowledge, these compositional HDPE systems have not been previously assessed.

## 2. Materials and Methods

### 2.1. Sample Preparation

**Figure 1:**
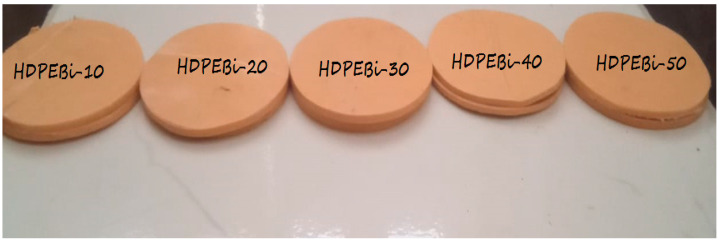
Picture of prepared bismuth oxide containing high-density polyethylene.

A quantity of high-density polyethylene was obtained from Sidi Kerir Petrochemicals Company, weighed with a 0.0001 g sensitive scale, and placed in a thermal mill at an effective temperature of 140 °C, where the melting point of polyethylene is 130 °C. The mill was operated at a rotation speed of 40 revolutions per minute (rpm) for a period of one-third of an hour. Powdered bismuth oxide was purchased from Al-Gomhoria Chemicals Company in Egypt with a purity of 98.7% and was filtered using a sieve having a diameter of 50 µm. After making sure that high-density polyethylene was completely melted, powdered bismuth oxide was gradually added to the specific amounts presented in Table 1, and to ensure that the mixture had become completely homogeneous, rotation was performed for a quarter of an hour. Then, the mixture was placed in a mold with dimensions of 125 × 125 × 30 mm, and the samples were pressed with a hydraulic heat press at a pressure of 10 MPa and a temperature of 200 °C for a quarter of an hour; the pressure was gradually increased to 20 MPa for another quarter of an hour. It remained under pressure to gradually cool using water at 20 °C, and at the end, the prepared sample was taken and cut into suitable discs to measure its shielding efficiency [24,25,26]. Figure 1 shows a picture of the prepared bismuth oxide containing high-density polyethylene. All experimental works were performed at Plastic Technology Center in Victoria, Egypt.

### 2.2. Morphological Test

Scanning electron microscopy (SEM) was used to analyze the microstructure of bismuth-injected high-density polyethylene samples to obtain the characterization of the samples. A JSM-5300 JEOL microscope was used [27].

### 2.3. Gamma Attenuation Test

**Figure 2:**
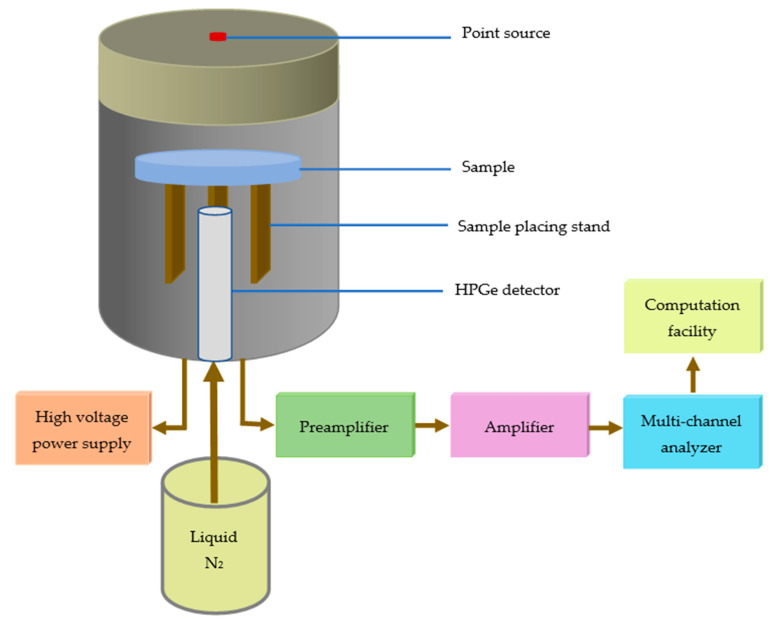
Illustration of setup of the experimental work.

An HPGe detector and three radioactive point sources were used to test the shielding parameters of the HDPE-Bi_2_O_3_ samples (see Figure 2). The details for the experimental measurement are given in References [28,29]. 

The experimental linear attenuation coefficient (LAC) was determined using the following equation [30,31]:

(1) $$\mathrm{LAC}=\frac{1}{t}\;ln\frac{N_{0}}{N}$$

The experimental results of the LACs of the HDPE-Bi_2_O_3_ samples were compared with the results obtained using Phy-X software [27]. The MFP and HVL were calculated based on LAC calculations [32,33,34,35,36,37].

## 3. Results and Discussion

### 3.1. Morphological Results

**Figure 3:**
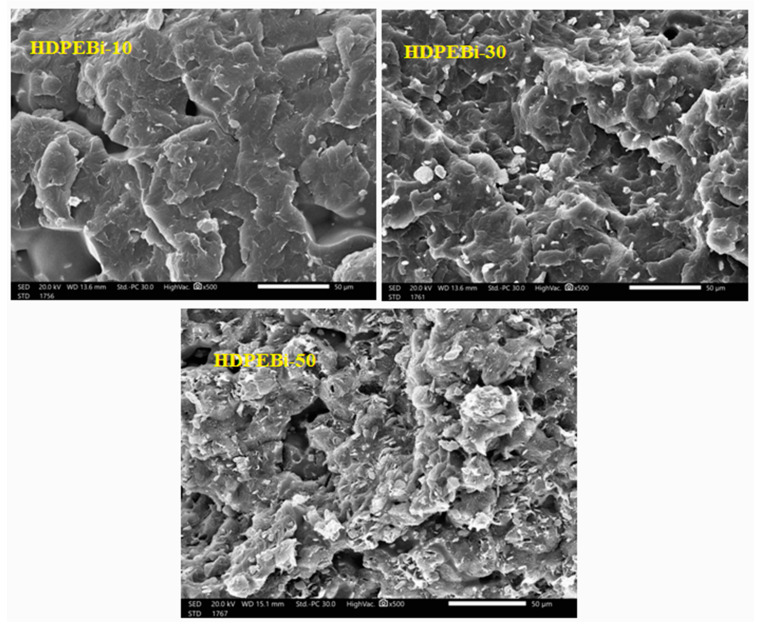
SEM images of different prepared HDPE-Bi_2_O_3_ samples.

Scanning was performed to know the distribution of bismuth oxide particles within HDPE using the electron microscope, and it was clear that the particles uniformly distributed and that the amount of Bi_2_O_3_ increased in the matrix with the increase in the proportion of particles, as shown in Figure 3. The higher the percentage of filler particles (Bi_2_O_3_) was, the more uniformed the distribution of particles inside the polymer was; therefore, the rate of photon collision with the material was higher due to the gaps being filled by Bi_2_O_3_ particles, and consequently, the attenuation of the incident photons was higher. We could conclude that the addition of bismuth particles improved the shielding properties of HDPE.

### 3.2. Gamma Attenuation Results

**Figure 4:**
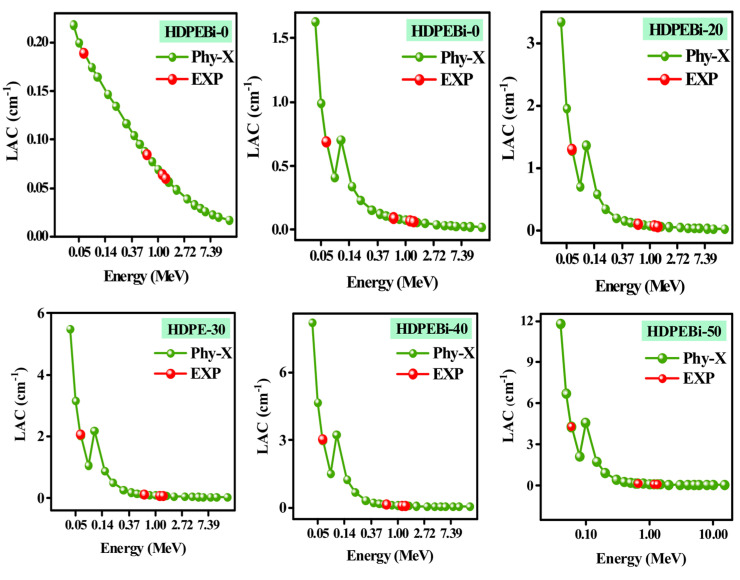
Linear attenuation coefficients of all prepared samples at different energies according to experimental and Phy-X results.

In this study, HDPEBi-10 (10 wt% Bi_2_O_3_), HDPEBi-20 (20 wt% Bi_2_O_3−_), HDPEBi-30 (30 wt% Bi_2_O_3_), HDPEBi-40 (40 wt% Bi_2_O_3_), and HDPEBi-50 (50 wt% Bi_2_O_3_) HDPE systems were prepared by adding Bi_2_O_3_ to HDPEBi-0 (pure HDPE). To compare the experimental results (obtained using HPGe) with the theoretical results (obtained using Phy-X software), the linear attenuation coefficients were measured in the lab at four different energies; their graphical representation is presented in Figure 4. The aim of this comparison was to validate the setup in this study, i.e., to check the accuracy of the experimental results. It is very clear from Figure 4 that from 0.06 MeV to 1.333 MeV, all the experimental values were in line with Phy-X software data, which validated the research setup.

**Figure 5:**
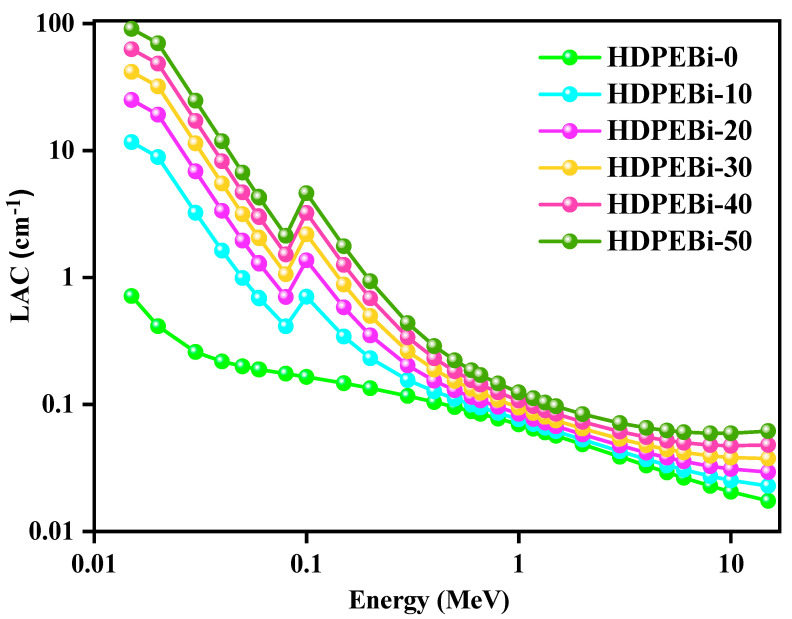
Linear attenuation coefficients of all HDPE systems at different energies according to PHY-X.

We calculated the LACs in a wide energy range to examine the behavior of the LACs at higher energies (see Figure 5). For all studied energies, the LAC values of the HDPE systems (containing Bi_2_O_3_) followed the following declining trend: HDPEBi-50 > HDPEBi-40 > HDPEBi-30 > HDPEBi-20 > HDPEBi-10. At all energy values E < 0.3 MeV, the HDPE systems supplemented with Bi_2_O_3_ showed higher LAC values. At the energy of 0.015 MeV, the values of the LACs of all studied HDPE systems were as follows: HDPEBi-0, 0.71 cm^−1^; HDPEBi-10, 11.7 cm^−1^; HDPEBi-20, 25 cm^−1^; HDPEBi-30, 41.6 cm^−1^; HDPEBi-40, 62.8 cm^−1^; and HDPEBi-40, 90.8 cm^−1^. HDPE system HDPEBi-50 (50 wt% Bi_2_O_3_) showed the highest LAC value compared with the other HDPE systems, which indicated that a higher amount of Bi_2_O_3_ in pure HDPE boosted the radiation shielding ability.

**Figure 6:**
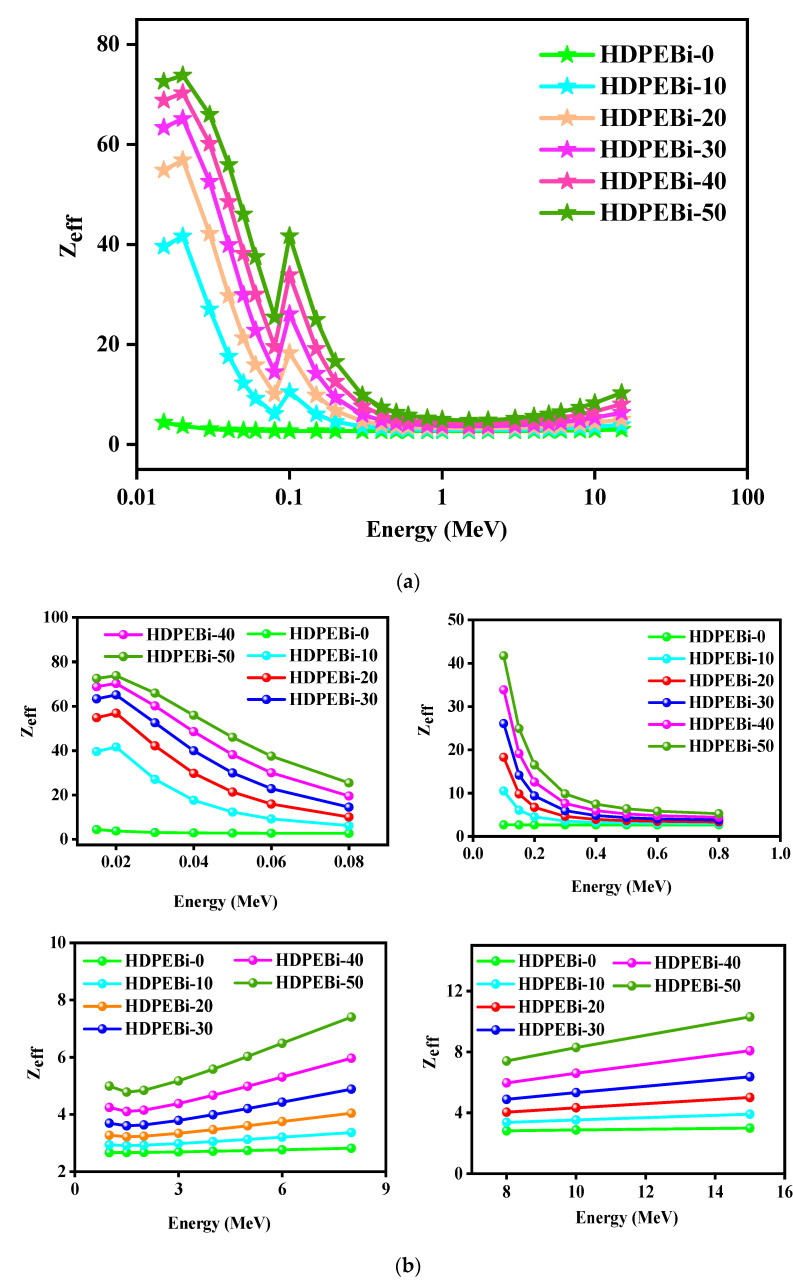
(**a**) Brief presentation of effective atomic numbers as functions of photon energy. (**b**) Detailed presentation of Z_eff_ as functions of photon energy.

HDPEBi-0, HDPEBi-10, HDPEBi-20, HDPEBi-30, HDPEBi-40, and HDPEBi-50 were HDPE series with diverse concentrations of Bi_2_O_3_, and the discrepancies among the assessed Z_eff_ values are presented as functions of the photon energy in Figure 6a. The values of Z_eff_ lay in the ranges of 4–3, 42–3, 57–3, 65–4, 70–4, and 74–5, respectively. The maximum values of the effective atomic number (Z_eff_) originated at the low energy of 0.015 MeV and were 4, 40, 55, 63, 69, and 73 for the studied HDPE series (HDPEBi-0, HDPEBi-10, HDPEBi-20, HDPEBi-30, HDPEBi-40, and HDPEBi-50, respectively). Yet, the Z_eff_ values of all studied HDPE systems followed a similar trend after Bi_2_O_3_ contamination, and HDPEBi-50 showed the highest Z_eff_ value, whereas HDPEBi-10 showed the lowest Z_eff_ value. Here, HDPEBi-10, HDPEBi-20, HDPEBi-30, HDPEBi-40, and HDPEBi-50 showed values 9, 13, 15, 16, and 17 times greater than that of HDPEBi-0 at the energy of 0.015 MeV thanks to the addition of Bi_2_O_3_ at 10, 20, 30, 40 and 50 wt% to HDPE. For energy values in the range of 0.02–0.08 MeV, Z_eff_ decreased and rapidly came down. It was clear that a higher amount of Bi_2_O_3_ increased the Z_eff_ value of HDPE. An exponential decrease was found for the energy range of 0.1–0.6 MeV; however, in the 1–15 MeV energy range, the effective atomic number (Z_eff_) sharply increased. In the energy range of 2–15 MeV, HDPEBi-10, HDPEBi-20, HDPEBi-30, HDPEBi-40, and HDPEBi-50 showed values 1.2, 1.5, 1.9, 2.4, and 3.0 times greater than that of HDPEBi-0. Here, the minimum values were seen at an energy of 1.5 MeV, and the values were 2.7, 2.9, 3.2, 3.6, 4.1, and 4.8 for the studied HDPE series, respectively (see Figure 6b).

**Figure 7:**
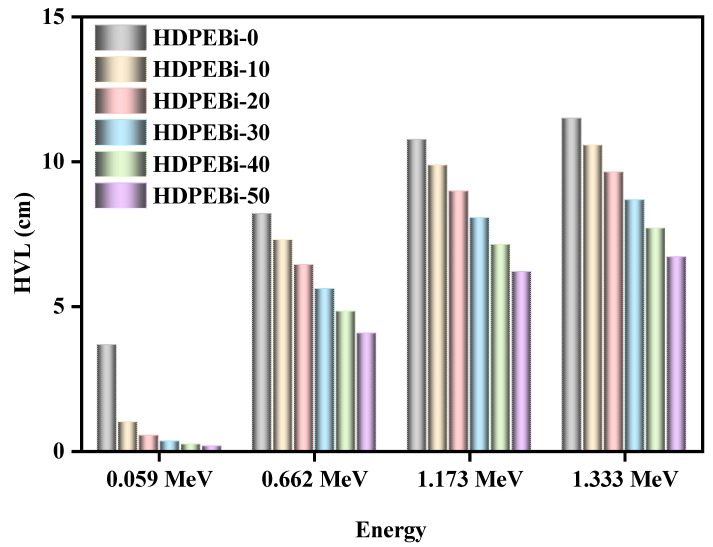
Half-value layer values as functions of photon energy (experimental results).

In Figure 7, the HVL values are plotted for the pure HDPE means without Bi_2_O_3_ (HDPEBi-0) and HDPE containing 10–50% Bi_2_O_3_ (HDPEBi-10, HDPEBi-20, HDPEBi-30, HDPEBi-40, and HDPEBi-50). The figure shows that the HVL of the studied samples increased with the increase in energy (this was correct for all compositions). From Figure 7, it is very clear that sample HDPEBi-0 showed an HVL value 1.7 times greater than that of HDPEBi-50 at the energy of 1.333 MeV, but at 0.0595 MeV, it was 23 times greater. That means that at very low energy values, HDPE containing 10–50% Bi_2_O_3_ could be used for protection from hazardous radiation, but with the increase in energy, the thickness of HDPE must be increased in order to obtain suitable protection from the high energy of radiation. It was revealed that at any fixed energy, the HVL decreased with the addition of Bi_2_O_3_ in HDPE. Pure HDPE showed a higher HVL than the other studied HDPE samples containing Bi_2_O_3_. Moreover, HDPEBi-50, with the highest content (50 wt%) of Bi_2_O_3_, showed the lowest HVL value. Thus, all studied HDPE samples containing Bi_2_O_3_ showed better radiation shielding competence than pure HDPE. Hence, it was clear that Bi_2_O_3_ addition was the cause of the reduction in the thickness of the HDPE samples that could attenuate the photon incidence.

**Figure 8:**
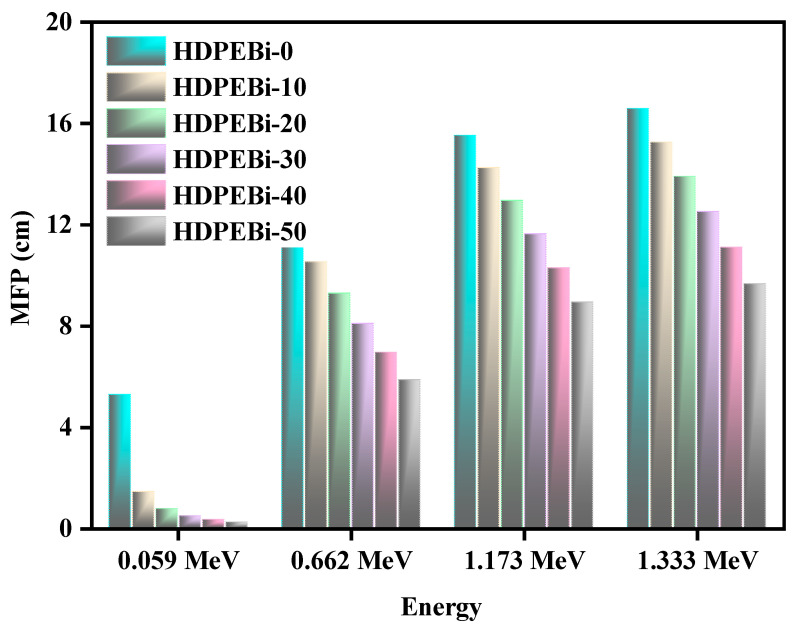
Mean free paths for all prepared samples at different energies.

To validate the efficacies of pure HDPE (without Bi_2_O_3_) and of the HDPE (with 10–50% Bi_2_O_3_) systems, the mean free path (MFP) values were examined herein for identifying the radiation shielding ability and the gained MFP fallouts for the pure and contaminated HDPE systems against photon energy, as demonstrated in Figure 8. This figure shows that Bi_2_O_3_-containing HDPE systems (HDPEBi-10, HDPEBi-20, HDPEBi-30, HDPEBi-40, and HDPEBi-50) showed lower MFP values than pure HDPE at the low energy levels of 0.0595 MeV and 0.0810 MeV. This provided the suggestion that these HDPE systems with the apt addition of Bi_2_O_3_ showed proficiency as radiation shielding materials. It is eminent that lower MFP values designate a healthier radiation shielding ability in any absorbing material. The MFP values of the HDPE systems ranked as follows: HDPEBi-50 < HDPEBi-40 < HDPEBi-30 < HDPEBi-20 < HDPEBi-10 < HDPEBi-0. The MFP values of HDPE systems HDPEBi-0, HDPEBi-10, HDPEBi-20, HDPEBi-30, and HDPEBi-40 were found to be 1.7, 1.6, 1.4, 1.3, and 1.2 times higher than that of HDPEBi-50 against Co-60 gamma irradiation. HDPE system HDPEBi-50 (50 wt% Bi_2_O_3_) had the lowermost MFP value among the other HDPE systems; hence, we could conclude that the radiation shielding features of the HDPE systems improved with the addition of Bi_2_O_3_.

## 4. Conclusions

Very few data on HDPE exist in terms of radiation shielding purposes, even though HDPE is extensively used worldwide. Hence, various amounts of Bi_2_O_3_ in HDPE were studied to identify its radiation shielding capability. The values of the linear attenuation coefficients obtained using Phy-X software and an HPGe detector were compared to ensure the sample preparation was consistent. The Z_eff_ maximum values originated at the low energy of 0.015 MeV and were 4, 40, 55, 63, 69, and 73 for the studied HDPE series, i.e., HDPEBi-0, HDPEBi-10, HDPEBi-20, HDPEBi-30, HDPEBi-40, and HDPEBi-50, respectively. HDPEBi-50 showed the highest Z_eff_ value, whereas HDPEBi-10 showed the lowest Z_eff_ value. Here, HDPEBi-10, HDPEBi-20, HDPEBi-30, HDPEBi-40, and HDPEBi-50 showed values 9, 13, 15, 16, and 17 times greater than that of HDPEBi-0 at the energy of 0.015 MeV. The MFP values of the HDPE systems ranked in the following order: HDPEBi-50 < HDPEBi-40 < HDPEBi-30 < HDPEBi-20 < HDPEBi-10 < HDPEBi-0. The MFP values of HDPE systems HDPEBi-0, HDPEBi-10, HDPEBi-20, HDPEBi-30, and HDPEBi-40 were found to be 1.7, 1.6, 1.4, 1.3, and 1.2-times higher than of HDPEBi-50 against Co-60 gamma irradiation. In the energy range of 0.015 MeV to 15 MeV, HDPE systems showed greater shielding ability according to their higher contents of Bi_2_O_3_.

## Figures and Tables

**Table 1:** Codes, chemical compositions, and densities of HDPE-Bi_2_O_3_ composites.

Code	Composition (wt%)		Density (g/cm^3^)
	HDPE	Bi_2_O_3_	
HDPEBi-0	100	-	0.959 ± 0.005
HDPEBi-10	90	10	1.053 ± 0.004
HDPEBi-20	80	20	1.167 ± 0.009
HDPEBi-30	70	30	1.310 ± 0.006
HDPEBi-40	60	40	1.491 ± 0.003
HDPEBi-50	50	50	1.731 ± 0.008

## Data Availability

All relevant data are within this paper.
